# Prevalence, Genetic Heterogeneity, and Antibiotic Resistance Profile of* Listeria* spp. and* Listeria monocytogenes* at Farm Level: A Highlight of ERIC- and BOX-PCR to Reveal Genetic Diversity

**DOI:** 10.1155/2018/3067494

**Published:** 2018-07-03

**Authors:** Lesley Maurice Bilung, Lai Sin Chai, Ahmad Syatir Tahar, Chong Kian Ted, Kasing Apun

**Affiliations:** ^1^Faculty of Resource Science and Technology, Universiti Malaysia Sarawak, 94300 Kota Samarahan, Sarawak, Malaysia; ^2^Institute of Biological Sciences, Faculty of Science, University of Malaya, 50603 Kuala Lumpur, Malaysia

## Abstract

This study aimed to identify* Listeria* spp. and* L. monocytogenes*, characterize the isolates, and determine the antibiotic resistance profiles of the isolates* Listeria* spp. and* L. monocytogenes* in fresh produce, fertilizer, and environmental samples from vegetable farms (organic and conventional farms). A total of 386 samples (vegetables, soil, water, and fertilizer with manure) were examined. The identification of bacterial isolates was performed using PCR and characterized using ERIC-PCR and BOX-PCR. The discriminating power of the typing method was analyzed using Simpson's Index of Diversity. Thirty-four (n=34)* Listeria *isolates were subjected to antimicrobial susceptibility test using the disc-diffusion technique. The PCR analysis revealed that* Listeria* spp. were present in 7.51% (29/386) of all the samples (vegetable, soil, fertilizer, and water). None of the samples examined were positive for the presence of* L. monocytogenes. * Percentages of 100% (15/15) and 73.30% (11/15) of the* Listeria* spp. isolated from vegetables, fertilizer, and soil from organic farm B had indistinguishable DNA fingerprints by using ERIC-PCR and BOX-PCR, respectively.* Listeria* spp. isolated from 86 samples of vegetable, fertilizer, and environment of organic farm A and conventional farm C had distinct DNA fingerprints. Simpson's Index of Diversity, D, of ERIC-PCR and BOX-PCR is 0.604 and 0.888, respectively. Antibiotic susceptibility test revealed that most of the* Listeria* spp. in this study were found to be resistant to ampicillin, rifampin, penicillin G, tetracycline, clindamycin, cephalothin, and ceftriaxone. The isolates had MAR index ranging between 0.31 and 0.85. In conclusion, hygienic measures at farm level are crucial to the reduction of* Listeria* transmission along the food chain.

## 1. Introduction

*Listeria* is a gram-positive, rod-shaped, and non-spore-forming bacterium [1]. Genus of* Listeria* is classified into 17 species including* Listeria monocytogenes *that is a common causative agent of human foodborne infection, listeriosis [2]. Listeriosis is usually treated with antibiotic therapy involving the use of penicillin, ampicillin, rifampin, gentamicin, tetracycline, erythromycin, chloramphenicol, or trimethoprim with sulfamethoxazole alone or in combination [3, 4]. Previous researches have shown that* Listeria* spp. may be resistant to several antibiotics such as clindamycin, daptomycin, oxacillin, tetracycline, and nalidixic acid [5, 6]. Therefore, it is important to monitor the antibiotic susceptibility of* Listeria* spp. and* L. monocytogenes* to ensure the effectiveness of listeriosis treatment.

Repetitive sequence-based PCR (Rep-PCR) is a DNA amplification technique for bacterial genomic fingerprinting by using repetitive DNA elements present within bacterial genome. There are four main types of repetitive sequences used for molecular typing which include enterobacterial repetitive intergenic consensus (ERIC) sequence, BOX elements, repetitive extragenic palindromic sequences (REP elements), and (GTG)_5_ [7]. Utilization interspersed repetitive sequence-based tools can be used in bacterial fingerprinting since the distance between each of the sequences varies among strains [8] and have been used to type wide range of gram-negative and several gram-positive bacteria [7]. ERIC-PCR has been used for intraspecies fingerprinting of* Bacillus anthracis* and* Bacillus cereus* [9],* Enterobacter sakazakii* [10],* Lactobacillus* [11],* Listeria monocytogenes* [12], and* Salmonella *Enteritidis [13, 14]. Meanwhile, BOX-PCR has been well used in typing of* Escherichia coli* [15–17],* Bifidobacterium* [7],* Streptomyces* [18],* Aeromonas* spp. [19],* Burkholderia pseudomallei *[20], and* Bacillus anthracis *[21]. Nonetheless, ERIC sequence-based PCR (ERIC-PCR) and BOX-PCR were used in this study as they are rapid subtyping methods and have high discrimination power [2, 22, 23].

According to Strawn et al. [24], agricultural practices (irrigation with contaminated water, fertilization with contaminated manure and contaminated soil) could increase the risk of bacterial contamination of vegetables. Therefore, this study was carried out to assess the contamination levels of* Listeria* spp. and* L. monocytogenes* in vegetables, fertilizer, and environmental samples (soil and water) at farm level practicing organic and conventional farming in Sarawak, to obtain information on the genetic diversity of the* Listeria* spp. isolates using Repetitive Intergenic Consensus Polymerase Chain Reaction (ERIC-PCR) and BOX-PCR. Further, the study aimed to compare the effectiveness of ERIC-PCR and BOX-PCR for genetic diversity of* Listeria* spp. and determine antibiotic resistance profiles of the* Listeria* spp. isolates.

## 2. Materials and Methods

### 2.1. Sampling Sites and Sample Collection

A total of 206 vegetable samples, 60 fertilizer samples, 60 soil samples, and 60 water samples were collected from two organic farms (organic farm A and organic farm B) and one conventional farm (conventional farm C) in Kuching, Sarawak. As shown in Table 1, the organic farms practice crop rotations, application of composted chicken waste and plant waste as fertilizer, mechanical methods to control weeds, and restricted use of pesticides. The conventional farm also practices the use of composted animal manures and plant waste as fertilizer; however, it does not practice crop rotation and synthetic chemical pesticides are applied to the produce biweekly.

The vegetable samples and soil samples were collected from the fields while the fertilizer samples were collected from the fertilizer storage places. Water samples were collected from the respective water sources (pond or rainwater). All samples were kept in ice box and transported to the Molecular Microbiology Laboratory at Universiti Malaysia Sarawak.

### 2.2. Sample Processing and Listeria Enrichment

Sample processing was done as described in Ozbey et al. [25]. First, 25 g (or 25 ml) of each sample (soil, fertilizer, and water) was weighed or measured and then transferred into conical flasks containing 225 ml of* Listeria* Enrichment Broth (LEB) (Oxoid, United States). After that, the flasks were incubated at 30°C for 48 hr. Vegetable samples were weighed (25 g) and cut into pieces before being transferred into the conical flasks containing 225 ml of LEB followed by incubation at 30°C for 48 hr.

### 2.3. Enumeration of Listeria spp.

The enriched bacterial cultures (100 *μ*l) from the samples were transferred and spread on PALCAM agar (Merck, Germany). The PALCAM agar plates were incubated at 37°C for 48 hr.* Listeria* spp. colonies appeared to be greyish-green or black in color and surrounded by black halo on PALCAM agar [26]. These colonies were observed, counted, and recorded.

### 2.4. DNA Extraction

Prior to amplification, genomic DNA was extracted using GF-1 Nucleic Acid Extraction Kits (Vivantis, United States) according to manufacturer's guide. DNA template was further subjected to PCR-based analysis.

### 2.5. Identification of Listeria spp. and Listeria monocytogenes

#### 2.5.1. DNA Extraction

Presumptive* Listeria* spp. colonies were selected from PALCAM agar and subjected to DNA extraction using GF-1 Nucleic Acid Extraction Kits (Vivantis, United States) according to the manufacturer's guide. DNA template was further subjected to PCR-based analysis.

#### 2.5.2. Polymerase Chain Reaction (PCR) for Listeria spp.

PCR detection of* Listeria* spp. was carried out as described by Jeyaletchumi et al. [27] and Wong et al. [28] with slight modification in the concentration of reagents. The primer pairs used for the detection of* Listeria* spp. (genus specific) were 5′-CTC CAT AAA GGT GAT CCT-3′ and 5′-CAG CAG CCG CGG TAA TAC-3′. These primers were designed to amplify a 938 bp region in the 16S rRNA gene. To prepare 25 *μ*l of PCR mixture, 0.2 M of forward primer, 0.2 M of reverse primer, 5.5 *μ*l of DNA template, 1.5 *μ*l of 10×* Taq *PCR buffer, 0.2 *μ*l dNTP, 1.5 mM MgCl_2_, and 1.5 unit of* Taq *DNA polymerase were mixed together. Lastly, the PCR products were separated on 1% agarose gel with 100 kb DNA ladder for 75 min, stained with ethidium bromide, and viewed under a UV transilluminator (Maestrogen, Taiwan).

#### 2.5.3. Polymerase Chain Reaction (PCR) for Listeria monocytogenes

PCR detection of* L. monocytogenes* was carried out as described by Awaisheh [29] with a modification in the concentration of reagents. The primer pairs used for the detection of* L. monocytogenes* were 5′-CAT TAG TGG AAA GAT GGA ATG-3′ and 5′-GTA TCC TCC AGA GTG ATC GA-3′ which amplify 730 bp region of the listeriolysin (*hlyA*) gene. To prepare 25 *μ*l of PCR mixture, 0.4 M of* hlyA* forward primer, 0.4 M of* hlyA* reverse primer, 5 *μ*l of DNA template, 2.5 *μ*l of 10×* Taq *PCR buffer, 0.2 mM dNTP, 0.8 mM MgCl_2_, and 2.5 units of* Taq *DNA polymerase were mixed together. Lastly, the PCR products were separated on 1% agarose gel with 100 kb DNA ladder for 75 min. The gel was stained with ethidium bromide and viewed under a UV transilluminator (Maestrogen, Taiwan).

### 2.6. Genetic Diversity Analysis Using ERIC and BOX-PCR

The ERIC-PCR condition for this method was in accordance with Indrawattana et al. [30] and Laciar et al. [31]. Meanwhile, the BOX-PCR condition followed Jamali and Thong [32] and Versalovic et al. [8] with slight modifications on the reagent concentration and reaction condition. In ERIC-PCR, the primer pairs used were 5′-ATGTAAGCTCCTGGGGATTCAC-3′ and 5′-AAGTAAGTGACTGGGGTGAGCG-3′. To prepare 25 *μ*l of PCR mixture, 1.0 M of forward primer, 1.0 M of reverse primer, 3.0 l of DNA template, 5.0 *μ*l of 5×Taq PCR buffer, 0.2 mM dNTP, 2.0 mM MgCl_2_, and 1.0 unit of* Taq* DNA polymerase were mixed together. The PCR reaction was carried out according to the condition in Table 2. In BOX-PCR, the primer used was BOX A1R (5′-CTACGG CAA GGC GAC GCT GAC G-3′). To prepare 25 *μ*l of PCR mixture, 400 *μ*M of each dNTPs, 1×PCR buffer, 3 mM MgCl_2_, 4 *μ*M of primer, and 2.5 U* Taq* DNA polymerase (Promega) were mixed together. The PCR reaction was carried out according to the condition in Table 3.

The PCR products from ERIC- and BOX-PCR were separated in 2% agarose gel with 100 kb DNA ladder for 90 min. Then, the gel was stained with ethidium bromide and viewed under a UV transilluminator (Maestrogen, Taiwan). The DNA band patterns were analyzed and a dendrogram was generated for the* Listeria* isolates by using BioNumerics 7.5 software program (Applied Maths, Sint-Martens-Latem, Belgium) using Dice coefficient and the unweighted pair group method (UPGMA) [33]. Simpson's Index of Diversity, D, was also calculated.

The discriminating power of this typing method was calculated by using Simpson's Index of Diversity, D [34]. The higher the discriminatory index, the greater the effectiveness of a particular fingerprinting method to discriminate different strains [35]. This index was given by the following equation:

(1) $$\begin{array}{c} D=1-\left(\;\frac{1}{N\left(N-1\right)}\;\right)\overset{s}{\underset{j=1}{\sum }}n_{j}\left(\;n_{j}-1\;\right) \end{array}$$

“*N*” denotes the total number of strains in the sample population, “*s*” denotes the total number of types described, “*n*_*j*_” denotes the number of strains belonging to the* j*th type.

### 2.7. Antibiotic Susceptibility Test

Antibiotic susceptibility of isolated* Listeria* spp. was carried out with the disc-diffusion method by Chen et al. [5] and Morobe et al. [36] with a slight modification. The antibiotic discs (Oxoid, the United States) used were ampicillin (10 *μ*g), cephalothin (30 *μ*g), chloramphenicol (30 *μ*g), clindamycin (2 *μ*g), erythromycin (15 *μ*g), gentamycin (10 *μ*g), penicillin G (10 *μ*g), rifampin (5 *μ*g), streptomycin (10 *μ*g), tetracycline (30 *μ*g), sulfamethoxazole/trimethoprim (23.75 *μ*g/1.25 *μ*g), novobiocin (30 *μ*g), nitrofurantoin (10 *μ*g), and ceftriaxone. First, the overnight culture grown in Mueller-Hinton broth was spread uniformly onto the Mueller-Hinton agar plate. Antibiotic discs were then placed onto the surface of each plate (4 antibiotics/Petri dish) using antibiotic-disc dispenser (Oxoid, United States). After incubation at 37°C for 24 hr, the diameter of growth inhibition zone surrounding each disc was measured and interpreted according to the CLSI (Clinical and Laboratory Standards Institute) 2014 recommendation. Evaluation of the* Listeria* as resistant, susceptible, and intermediate toward the antibiotics was conducted by referring to the Zone Diameter Interpretive Criteria (nearest whole mm) of a particular antibiotic of CLSI. The CLSI criteria for staphylococci were referred to in this study because interpretative criteria for* Listeria* are not available from CLSI with the exception of susceptibility breakpoints for ampicillin and penicillin. Multiple antibiotic resistance (MAR) index of an isolate was calculated as defined by Krumperman [37]:

(2) $$\begin{array}{c} MAR\;\;index=\frac{a}{b} \end{array}$$

*“a”* denotes number of antibiotics to which the particular isolate was resistant and* “b”* denotes number of antibiotics to which the particular isolate was exposed.

## 3. Results

### 3.1. Prevalence of Listeria spp. Based on PCR Analysis

**Figure 1:**
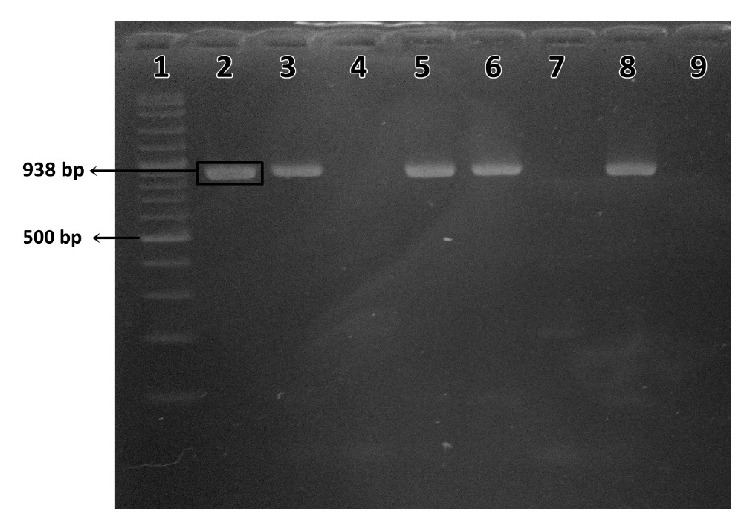
PCR amplification of 16S rRNA gene of* Listeria* spp. with the expected size of 938 bp, in fertilizer samples obtained from organic farm B. Lane 1: 100 bp ladder. Lane 2:* L. monocytogenes *reference strains ATCC 19155. Lanes 3-8: presumptive* Listeria* spp. isolates from fertilizer samples. Lane 9: negative control.

Analysis using PCR assay revealed that* Listeria* spp. were present in 7.51% (29/386) of all the samples (vegetable, soil, fertilizer, and water) collected. It was present in 9.10% (6/66), 8.13% (13/160), and 6.25% (10/160) of the samples collected from organic farm A, organic farm B, and conventional farm C, respectively. The prevalence of* Listeria* spp. from all the samples was shown in Table 4. The gel picture for PCR amplification of 16S rRNA gene of* Listeria* spp. was shown in Figure 1.

### 3.2. Enumeration of Listeria spp. in the Vegetables, Soil, Water, and Fertilizer

The standard plate count (in CFU/g) of* Listeria* spp. in all the samples is also shown in Table 2.* Listeria* spp. were present in 6.70% (2/30) and 8.00% (7/88) of vegetable samples from organic farm A and organic farm B, respectively. Vegetable samples from organic farm B had the highest prevalence of* Listeria* spp. among the three farms, while soil samples from organic farm A have the highest prevalence of* Listeria* spp.* Listeria* spp. were not present in vegetables and soil samples from conventional farm C. For fertilizer samples, organic farms A and B had the highest prevalence of* Listeria* spp. among the three farms. For water samples,* Listeria* spp. were only present in 33.30% (8/24) of water samples from conventional farm C.

### 3.3. Prevalence of Listeria monocytogenes Based on PCR Analysis

PCR detection of* L. monocytogenes* in the samples was carried out by using primer pairs which amplified 730 bp region of the listeriolysin (*hlyA*) gene. However,* L. monocytogenes* was absent in all the samples analyzed.

### 3.4. Genetic Diversity of Listeria Isolates Using ERIC-PCR

**Figure 2:**
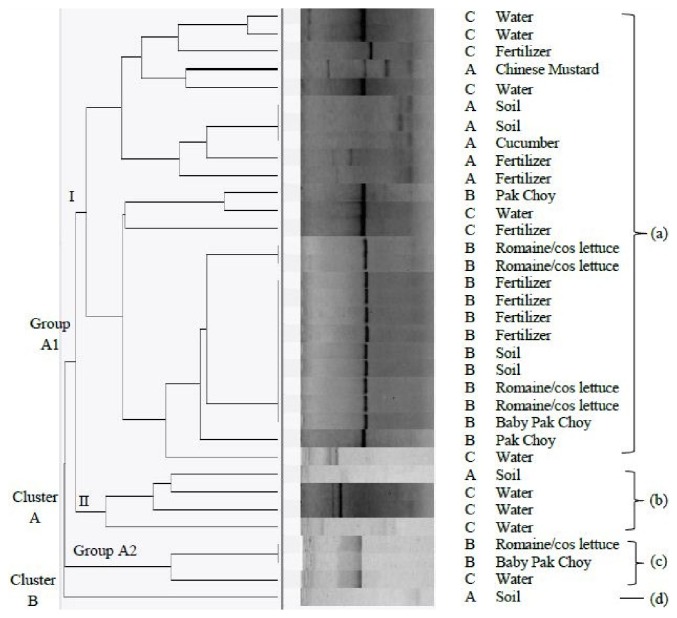
Dendrogram constructed for ERIC-PCR of* Listeria *spp. in vegetable, fertilizer, and environmental samples collected from organic farm A, organic farm B, and conventional farm. “A” denotes organic farm A, “B” denotes organic farm B, “C” denotes organic farm C, “(a)” denotes* Listeria *spp. isolated from vegetables, soil, fertilizer, and water from organic farm A, organic farm B, and conventional farm C, “(b)” denotes* Listeria* spp. isolated from soil and water from organic farm A and conventional farm C, “(c)” denotes* Listeria *spp. isolated from vegetable and water from organic farm B and conventional farm C, and “(d)” denotes* Listeria* spp. isolated from soil from organic farm A.

The electrophoretic profile of DNA fragments obtained after ERIC-PCR amplification yielded 1-5 bands with size approximately 120 bp to 1450 bp. A common band with molecular size of approximately 520 bp was observed in the electrophoretic profile from most of the isolates. Based on the ERIC-PCR dendrogram shown in Figure 2, the* Listeria* spp. isolated from organic farm A, organic farm B, and conventional farm C were genetically diverse and heterogeneous as they were not classified into specific cluster by either sampling area or the type of samples. ERIC-PCR analysis produced 11 different DNA fingerprint profiles. Simpson's Index of Diversity, D, was calculated for ERIC-PCR based on Hunter and Gaston [34]. The D value of this technique was calculated to be 0.604.

### 3.5. Genetic Diversity of Listeria Isolates Using BOX-PCR

**Figure 3:**
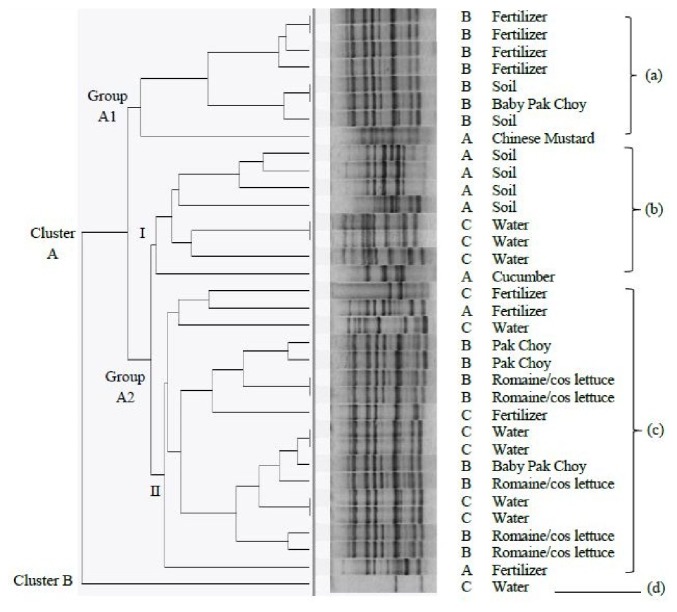
Dendrogram constructed for BOX-PCR of* Listeria* spp. in vegetable, fertilizer, and environmental samples collected from organic farm A, organic farm B, and conventional farm C. “A” denotes organic farm A, “B” denotes organic farm B, “C” denotes organic farm C, “(a)” denotes* Listeria *spp. isolated from vegetables, soil, and fertilizer from organic farm A and organic farm B, “(b)” denotes* Listeria *spp. isolated from vegetable, soil, and water from organic farm A and conventional farm C, “(c)” denotes* Listeria *spp. isolated from vegetables, fertilizer, and water from organic farm A, organic farm B, and conventional farm C, and “(d)” denotes* Listeria* spp. isolated from water from conventional farm C.

The electrophoretic band pattern of BOX-PCR amplification yielded 2-13 bands with size approximately 120 bp to 1550 bp. Based on the BOX-PCR dendrogram shown in Figure 3,* Listeria* spp. isolated from all the three farms were not classified according to the types of sample or sampling area. Therefore, these* Listeria* spp. isolates were genetically diverse and heterogeneous. BOX-PCR analysis produced 14 different fingerprint profiles. Simpson's Index of Diversity, D, was calculated for BOX-PCR based on Hunter and Gaston [34]. The D value of this technique was calculated to be 0.888.

### 3.6. Antibiotic Susceptibility Test

Thirty-four (n=34)* Listeria* spp. isolated from 29 samples (vegetable, soil, fertilizer, and water) collected from all the three farms were subjected to antibiotic susceptibility testing.* Listeria* spp. isolates were most resistant to clindamycin 97.06% (33/34) and least resistant to gentamicin 17.65% (6/34).

**Figure 4:**
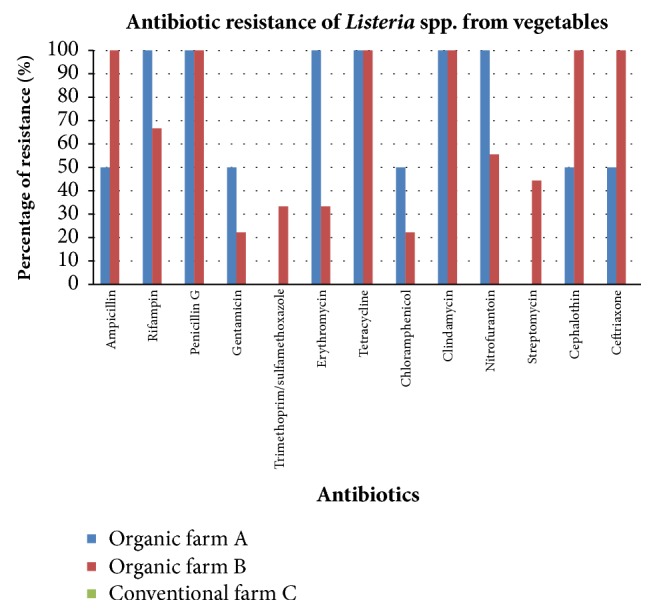
Percentage of antibiotic resistance of* Listeria* spp. from vegetable samples from organic farm A, organic farm B, and conventional farm C.

*Listeria* spp. were isolated from 11 vegetable samples, 2 (Chinese mustard and cucumber) from organic farm A and 9 (Chinese cabbage, romaine/cos lettuce, and Chinese white cabbage) from organic farm B. Antibiotic resistance graph of vegetable samples from the three farms is shown in Figure 4.

**Figure 5:**
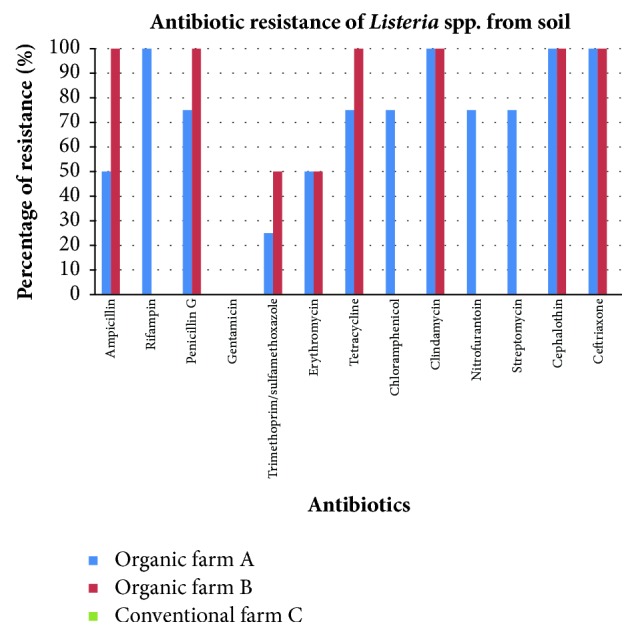
Percentage of antibiotic resistance of* Listeria* spp. from soil samples from organic farm A, organic farm B, and conventional farm C.

All* Listeria* spp. isolated from vegetable samples from organic farms A and B were resistant to penicillin G, tetracycline, and clindamycin. For soil samples,* Listeria* spp. were isolated from 6 soil samples, 4 from organic farm A and 2 from organic farm B. Antibiotic resistance graph of soil samples from the three farms is shown in Figure 5.

**Figure 6:**
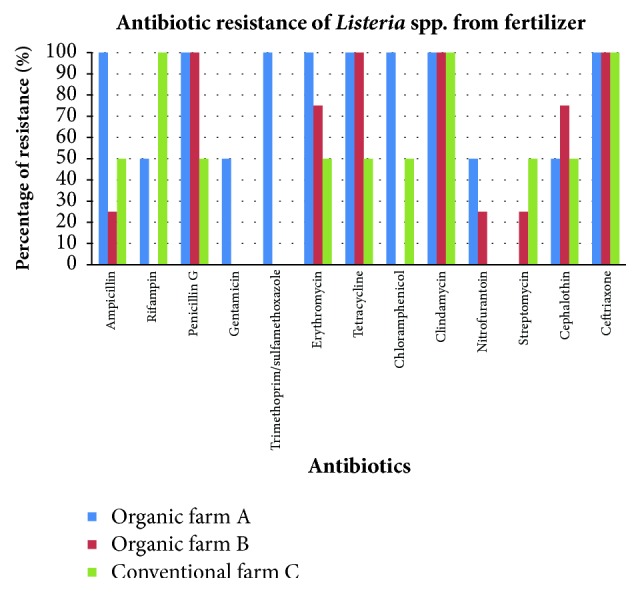
Percentage of antibiotic resistance of* Listeria* spp. from fertilizer samples from organic farm A, organic farm B, and conventional farm C.

In this study, all* Listeria* spp. isolated from soil samples from organic farms A and B were resistant to clindamycin, cephalothin, and ceftriaxone. Two* Listeria *spp., 4* Listeria *spp., and 2* Listeria *spp. were isolated from the fertilizer samples collected from organic farm A, organic farm B, and conventional farm C, respectively. Antibiotic resistance graph of fertilizer samples from the three farms is shown in Figure 6.

The results revealed that all* Listeria* spp. isolated from fertilizer samples were resistant to clindamycin and ceftriaxone. Conventional farm C was the only farm where the water samples were detected with* Listeria* spp., with 9* Listeria* spp. isolated. Antibiotic resistance graph of water samples from the 3 farms is shown in Figure 5. All (100%) (9/9) of the* Listeria* spp. were resistant to penicillin G. MAR index defined by Krumperman [37] was evaluated for all the isolates. In this study,* Listeria *spp. isolates demonstrated MAR; they were resistant to at least four of the thirteen antibiotics tested. The MAR indexes for all the isolates are recorded in Table 5. For vegetable samples,* Listeria *spp. in Chinese mustard from organic farm A and romaine/cos lettuce from organic farm B had the highest MAR index of 0.85. For soil samples,* Listeria* spp. from organic farm A had the highest MAR index of 0.69. For fertilizer samples,* Listeria *spp. from organic farm A had the highest MAR index of 0.85. For water samples,* Listeria* spp. were isolated only from conventional farm C, and the highest MAR index was 0.77.

## 4. Discussion

### 4.1. Prevalence of Listeria spp. from the Vegetables, Soil, Water, and Fertilizer

As shown in Table 4, a total of five vegetables from organic farms A and B had high concentration of* Listeria* spp. in the vegetables (ranging from 9.50 × 10^2^ to 2.10 × 10^5^ CFU/g). However, none of vegetables from conventional farm C was positive. According to the Public Health England [38], samples consisting of more than 100 CFU/g of* Listeria* spp. are considered unsatisfactory and investigation is required. Therefore, the vegetable samples collected from organic farms A and B were considered unsatisfactory and this represents the risk of contracting listeriosis associated with fresh produce consumption.* Listeria* spp. were present in 16.70% (2/12) and 8.30% (2/24) of soil from organic farms A and B.* Listeria* spp. are widely distributed in the environment including soil, vegetation, surface water, sewage, animal feeds, farm environments, and food-processing environments [39]. According to Vackachan et al. [40], contaminated fertilizer and humidity of the soil may increase the risk of soil contamination. Therefore, measures should be taken for the use of contaminated soil to reduce the presence of the bacteria. The fertilizer used by the three farms in the present study was animal waste compost (chicken litter) and plant waste. Such fertilizers are usually used as they are of low cost, organic, and containing notable amount of nutrients. Normally, composting of animal waste can inactivate large populations of human pathogens but improper composting or cross-contamination results in the high survival rate of these pathogens. Improper composting may also result in the regrowth of the pathogens in the finished compost products under a range of favorable conditions [41].* Listeria* spp. were absent in the water samples from organic farms A and B. However,* Listeria* spp. were detected in 33.30% (8/24) of the water from conventional farm C. According to Galvez et al. [42] and Chitarra et al. [43], pathogenic bacteria such as* Salmonella*, pathogenic* E. coli*, and* L. monocytogenes* can be found in irrigation water for fresh produce. These pathogenic bacteria can internalize crops through the roots and survive in them. This study also revealed no presence of* L. monocytogenes* in all the samples (vegetable, soil, water, and fertilizer) from all farms which could indicate lower potential of disease burden as the species is commonly causing human infections [2].

### 4.2. Genetic Heterogeneity of Listeria spp. Based on ERIC- and BOX-PCR Analysis

The findings from both ERIC-PCR and BOX-PCR analysis in the present study showed that the* Listeria* spp. isolates were not grouped together based on the types of samples and the source of isolation. They were not classified into specific cluster by either sampling area or the type of samples. In the present study, the* Listeria* spp. isolated from organic farm A, organic farm B, and conventional farm C were genetically diverse and heterogeneous. The heterogeneity was expected as the isolates were collected from different types of sample (vegetable, soil, fertilizer, and water) and sampling locations (organic farm A, organic farm B, and conventional farm C). In this study, Simpson's Index of Diversity, D, value for ERIC- and BOX-PCR was 0.604 and 0.888, respectively. According to Kqueen et al. [35], the higher the discriminatory index, the greater the effectiveness of a particular fingerprinting method to discriminate different strains. Thus, it was shown that BOX-PCR had greater discriminatory power than ERIC-PCR for fingerprinting* Listeria* spp. isolates of this study. In comparison, the discriminatory power for both BOX-PCR and ERIC-PCR analysis was lower as compared to the finding by Jersek et al. [44] which revealed ERIC-PCR was suitable for the typing of* L. monocytogenes* isolates as the index of discrimination was high (0.98). Another study conducted by Jamali and Thong [32] reported that the discrimination indexes for REP-PCR, BOX-PCR, RAPD, and PFGE were 0.992, 0.998, 1, and 0.916, respectively. They suggested that different subtyping methods often give different discriminatory powers. Therefore, it is necessary to use more than one subtyping approach to provide a more accurate description of the genetic diversity of microorganisms in the study. On the other hand, other fingerprinting tools such as REP-PCR and (GTG)_5_ are well employed in bacterial typing [8] which can be tested in further study.

### 4.3. Antibiotic Susceptibility of the Listeria Isolates

This present study revealed that 97.06% (33/34) of* Listeria *spp. isolated from vegetables, soil, fertilizer, and water from organic farm A, organic farm B, and conventional farm C were resistant to clindamycin. Chen et al. [5] found that all* Listeria *spp. isolates in catfish fillets and processing environment were resistant to clindamycin. Gamboa-Marin et al. [45] also revealed that* L. monocytogenes*,* Listeria* spp., and* L. ivanovii* from swine processing facilities in Colombia had major resistance and intermediate susceptibility to clindamycin. In the present study,* Listeria* spp. from the three farms showed the lowest resistance against gentamicin. This is comparable to a study by Li et al. [46] which revealed gentamicin exhibited good activity against* Listeria* spp. from processed bison in the USA.

This study showed that all the* Listeria* spp. isolates were resistant to more than one antibiotic and therefore demonstrated MAR. According to Krumperman [37], MAR index value lower than 0.20 indicates that the organism originated from a lower risk source in which the antibiotics are seldom or never used. MAR index value higher than 0.20 indicates that they are originated from a higher risk source which is greatly exposed to antibiotics. A study conducted by Jamali et al. [47] reported that 8.40% of* Listeria* spp. isolated from raw milk in Iran showed multiple antibiotic resistances. In the present study, all* Listeria* spp. had MAR index higher than 0.20, suggesting that the* Listeria* spp. isolates from the three farms were originated from a higher risk source in which they had been constantly exposed to antibiotics. MAR of* Listeria* spp. in vegetables, soil, and irrigation water could be a result of the usage of animal waste as fertilizer which might contain antibiotics used to prevent or treat animal diseases and promote animal growth. Hu et al. [48] conducted a study on the migration of antibiotics from manure to soil and from soil to vegetables and groundwater. In the study, they applied manure containing antibiotics to organic vegetable bases and revealed that the soil, vegetables, and water were detected with antibiotic residues. Some antibiotics are still biologically active despite being in environment. This eventually can initiate development of antibiotics resistance genes in microorganisms. The findings in the present study showed that* Listeria* spp. isolated from the samples from all the three farms were multiresistant to the antibiotics tested. The presence of* Listeria* spp. that were resistant to antibiotics commonly used to treat human listeriosis (including ampicillin, chloramphenicol, erythromycin, gentamicin, penicillin G, rifampin, tetracycline, and sulfamethoxazole/trimethoprim) raises the possibility of future acquisition of resistance by* L. monocytogenes* and* Listeria* spp.

## 5. Conclusion

The findings of this study show current occurrence of* Listeria* spp. at farm level of selected organic and conventional vegetable farms in Sarawak, Malaysia. Personal hygiene and good manufacturing practice at farm level are essential for prevention of the transmission of the organism along the food chain. Based on the genotyping analysis, all* Listeria* spp. isolates were heterogenous. Nonetheless, BOX-PCR was shown to be better in discriminatory power than ERIC-PCR and can be utilized in* Listeria* typing. This study also presented high occurrence of multiple antibiotic resistant strains in the fresh produce and farm environment which could be an indicator of the excessive use of antibiotics in the agriculture field.

## Figures and Tables

**Table 1:** Sampling period and agricultural practices for respective sampling sites.

Sampling site	Agricultural practices	Date of sampling trips
Organic Farm A	(i) Crop rotations (ii) Organic fertilizer (iii) Mechanical weed control (iv) No pesticides	19/02/14 13/03/14 24/03/14 21/04/14

Organic Farm B	(i) Crop rotations (ii) Organic fertilizer (iii) Mechanical weed control (iv) No pesticides	11/08/14 13/10/14 27/10/14 17/11/14

Conventional Farm C	(i) No crop rotation (ii) Organic fertilizer (iii) Application of synthetic pesticides	12/05/14 09/06/14 30/06/14 14/07/14

**Table 2:** PCR conditions for ERIC-PCR.

PCR steps	Temperature (°C)	Duration (min)	Cycle
Initial denaturation	95	5.00	-

Denaturation	90	0.50	30
Annealing	50	0.50	
Elongation	52	1.00	

Final Extension	72	8.00	-

**Table 3:** PCR conditions for BOX-PCR.

PCR steps	Temperature (°C)	Duration (min)	Cycle
Initial denaturation	94	5.00	-

Denaturation	94	1.00	35
Annealing	40	2.00	
Elongation	72	2.00	

Final Extension	72	10.00	-

**Table 4:** Prevalence of *Listeria* spp. in the organic farms and conventional farm by PCR assay.

No.	Types of sample	Organic farm			Conventional farm		
		Organic farm A	Standard Plate Count (CFU/g)	Organic farm B	Standard Plate Count (CFU/g)	Conventional farm C	Standard Plate Count (CFU/g)
1	Chinese cabbage (Pak Choy)	0% (0/6)	-	14.30% (2/14)	9.50 × 10^2^ -7.70 × 10^3^	0% (0/10)	-

2	Lettuce	0% (0/6)	-	-	-	0% (0/12)	-

3	Cucumber	16.70% (1/6)	2.10 × 10^5^	-	-	0% (0/14)	-

4	Yardlong bean	0% (0/4)	-	-	-	0% (0/14)	-

5	Chinese flowering cabbage (Choy Sum)	0% (0/3)	-	0% (0/16)	-	0% (0/10)	-

6	Tomato	0% (0/3)	-	-	-	0% (0/8)	-

7	Chinese Mustard	100% (1/1)	2.50 × 10^4^	0% (0/12)	-	0% (0/10)	-

8	Romaine/cos Lettuce	0% (0/1)	-	25% (4/16)	2.10 × 10^3^ - 9.90 × 10^4^	-	-

9	Chinese Kale (Kailan)	-	-	0% (0/8)	-	0% (0/10)	-

10	Chinese white cabbage (Sawi Manis)	-	-	6.30% (1/16)	1.30 × 10^4^	-	-

11	Local vegetable (Sabi Sative)	-	-	0% (0/6)	-	-	-

12	Soil	16.70% (2/12)	3.30 × 10^3^ -6.90 × 10^4^	8.30% (2/24)	1.00 × 10^5^ -1.20 × 10^5^	0% (0/24)	-

13	Fertilizer	16.70% (2/12)	1.20 × 10^4^ -2.20 × 10^4^	16.70% (4/24)	5.10 × 10^4^ -7.00 × 10^5^	8.30% (2/24)	4.30 × 10^4^ -1.50 × 10^5^

14	Water	0% (0/12)	-	0% (0/24)	-	33.30% (8/24)	1.00 × 10^3^ -3.50 × 10^6^

Total		9.10% (6/66)		8.13% (13/160)		6.25% (10/160)	

“ – “ denotes not available.

**Table 5:** MAR index for all *Listeria* spp. isolates from organic farm A, organic farm B, and conventional farm C.

No	Source	Sample	*Listeria* spp. Isolates	Antibiotic Resistance Pattern	MAR index
1	Organic Farm A	Chinese Mustard	V9b	Amp, Rif, PenG, Gen, Ery, Tet, Chl, Cf, Cli, Nor, Cro	0.85
2		Cucumber	V11a	Rif, PenG, Ery, Tet, Cli, Nor	0.46
3		Soil	S4a	Amp, Rif, PenG, Tet, Str, Cf, Cli, Nor, Cro	0.69
4			S4b	Rif, PenG, Sxt, Tet, Chl, Str, Cf, Cli, Cro	0.69
5			S4e	Rif, PenG, Ery, Chl, Cf, Cli, Nor, Cro	0.61
6		Soil	S5b	Amp, Rif, Ery, Tet Chl, Str, Cf, Cli, Nor, Cro	0.77
7		Fertilizer	M4a	Amp, Rif, PenG, Gen, Stx, Ery, Tet, Chl, Cf, Cli, Cro	0.85
8		Fertilizer	M5b	Amp, PenG, Sxt, Ery, Tet, Chl, Cf, Cli, Nor, Cro	0.77

9	Organic Farm B	Pak Choy	V114a	Amp, Rif, PenG, Tet, Cf, Cli, Nor, Cro	0.62
10		Pak Choy	V116c	Amp, PenG, Ery, Tet, Cf, Cli, Nor, Cro	0.62
11		Romaine/cos lettuce	V118a	Amp, Rif, PenG, Gen, Ery, Tet, Cf, Cli, Cro	0.69
12		Romaine/cos lettuce	V118c	Amp, Rif, PenG, Gen, Tet, Cf, Cli, Nor, Cro	0.69
13		Romaine/cos lettuce	V120b	Amp, Rif, PenG, Sxt, Tet, Cf, Cli, Nor, Cro	0.69
14		Romaine/cos lettuce	V120e	Amp, PenG, Sxt, Ery, Tet, Chl, Str, Cf, Cli, Nor, Cro	0.85
15		Sawi Manis	V120g	Amp, Rif, PenG, Tet, Chl, Str, Cf, Cli, Cro	0.69
16			V123e	Amp, Rif, PenG, Sxt, Ery, Tet, Str, Cf, Cli, Cro	0.77
17			V123f	Amp, PenG, Ery, Tet, Str, Cf, Cli, Cro	0.62
18		Soil	S37a	Amp, PenG, Sxt, Tet, Cf, Cli, Cro	0.54
19		Soil	S37c	Amp, PenG, Ery, Tet, Cf, Cli, Cro	0.54
20		Fertilizer	M38a	PenG, Ery, Tet, Cf, Cli, Nor, Cro	0.54
21		Fertilizer	M38f	PenG, Tet, Cf, Cli, Cro	0.38
22		Fertilizer	M39b	PenG, Ery, Tet, Cf, Cli, Cro	0.46
23		Fertilizer	M39f	Amp, PenG, Ery, Tet, Str, Cli, Cro	0.54

24	Conventional Farm C	Fertilizer	M16b	Amp, Rif, PenG, Str, Cf, Cli, Cro	0.54
25		Fertilizer	M22b	Rif, Ery, Tet, Chl, Cli, Cro	0.46
26		Water	W21b	Rif, PenG, Gen, Tet	0.31
27		Water	W23a	PenG, Tet, Cf, Cli, Cro	0.38
28		Water	W23b	Amp, Rif, PenG, Tet, Str, Cf, Cli, Nor, Cro	0.69
29		Water	W24a	Amp, Rif, PenG, Sxt, Tet, Chl, Str, Cf, Cli, Cro	0.77
30		Water	W24b	PenG, Tet, Chl, Cli, Nor, Cro	0.46
31			W24c	Amp, PenG, Gen, Sxt, Ery, Tet, Chl, Cf, Cli	0.69
32		Water	W26a	Amp, Rif, PenG, Tet, Str, Cli, Cro	0.54
33		Water	W27a	PenG, Sxt, Ery, Chl, Cli, Cro	0.46
34		Water	W27c	Amp, PenG, Sxt, Tet, Str, Cli, Nor, Cro	0.62

“Amp” denotes ampicillin, “Cf” denotes cephalothin, “Cro” denotes chloramphenicol, “Sli” denotes clindamycin, “Ery” denotes erythromycin, “Gen” denotes gentamycin, “PenG” denotes penicillin G, “Rif” denotes rifampin, “Str” denotes streptomycin, “Tet” denotes tetracycline, “Sxt” denotes sulfamethoxazole/trimethoprim, “Nor” denotes nitrofurantoin, and Cf denotes ceftriaxone.

## Data Availability

Data generated in this study are included in this article.
